# The role of PD-L1 in the radiation response and clinical outcome for bladder cancer

**DOI:** 10.1038/srep19740

**Published:** 2016-01-25

**Authors:** Chun-Te Wu, Wen-Cheng Chen, Ying-Hsu Chang, Wei-Yu Lin, Miao-Fen Chen

**Affiliations:** 1 Department of Urology, Chang Gung Memorial Hospital at Keelung, Taiwan; 2 Chang Gung University, College of Medicine, Taoyuan, Taiwan; 3 Department of Radiation Oncology, Chang Gung Memorial Hospital at Chiayi, Taiwan; 4 Department of Urology, Chang Gung Memorial Hospital at Linko, Taiwan; 5 Urology, Chang Gung Memorial Hospital at Chiayi, Taiwan

## Abstract

Identification of potential factors that can stratify a tumor’s response to specific therapies will aid in the selection of cancer therapy. The aim was to highlight the role of programmed cell death 1 ligand 1 (PD-L1) in bladder cancer. In this study, 92 of muscle-invasive bladder cancers and 28 of non- muscle invasive bladder cancers were selected for immunohistochemical staining analysis. Furthermore, human and murine bladder cancer cell lines were used to examine the correlation between PD-L1 and radiation response. Our data revealed that PD-L1 was overexpressed in the bladder tumor specimens compared with adjacent non-malignant specimens. Furthermore, the staining of PD-L1 was significantly linked to higher clinical stage, lower complete response rates and reduced disease-free survival rates. By *in vitro* and *in vivo* experiments, irradiation up-regulated the expression of PD-L1 in tumor cells, and its increase correlated with the irradiation dose. In immunocompetent mouse models, blocking PD-L1 induced a longer tumour growth delay following irradiation. The inhibition of T cell functions including proliferation and cytotoxicity against tumor cells was responsible to the effects of PD-L1 on radiation response. In conclusion, PD-L1 could be a significant clinical predictor for clinical stage and treatment response of bladder cancer.

Bladder cancer is a significant public health issue worldwide and manifests itself in two distinct forms with different clinical and biological behaviors^1^. Approximately 70% of patients presented with non-muscle-invasive bladder cancer (NMIBC) with good prognosis, and the remaining 30% with muscle-invasive disease has an unfavorable prognosis, with a 5-year recurrence-free survival estimated of about 60%^2^. Radical cystectomy with lymph node dissection is the gold standard for muscle invasive bladder cancer (MIBC), with an undeniable impact on urinary and sexual function. For bladder sparing, trimodality therapy (chemotherapy and concurrent radiation therapy following a complete TURBT) has been investigated as a strategy with approximately 50% long term disease-free survival reported in appropriately selected patients^3^,^4^. On the basis of the clinical data, this study was undertaken to determine the potential molecular markers that can increase the ability to predict which patients will response to CCRT and disease recurrence for patients with muscle-invasive bladder cancer.

Tumor-induced immune suppression in cancer patients is a major issue that not only promotes tumor progression but also inhibits the efficiency of anti-cancer treatment^5^,^6^. Radiotherapy (RT) engages host immune effector mechanisms that may contribute to the control and/or eradication of cancer^7^,^8^. However, radiation may be insufficient to generate an immune response that inhibits long-term relapse. Therefore, the identification and inhibition of key drivers of immunosuppression have the potential to improve patient outcome and increase treatment response. One of the major molecular regulators of tumor immune escape is programmed cell death 1 ligand 1 (PD-L1). PD-L1, a 40-kDa transmembrane protein belonging to the B7 family, negatively regulates T-cell signaling and inhibits T cell–mediated immune attack through binding to its receptor PD-1 on tumor-specific T cells^9^,^10^. PD-L1 has been reported to be over-expressed in several human malignancies and link to poor prognosis and the resistance to anticancer therapies^11^,^12^,^13^,^14^,^15^. The issue to explore the key targets that can block PD-L1 expression and then enhance T-cell function in cancers has been brought into spotlight. To date, preclinical and clinical evidence have suggested the augmentation of systemic antitumour immunity following local RT in combination with immunotherapy for cancers^16^,^17^,^18^. However, the specific mechanisms and appropriate patient populations required to examine the combinatorial treatment have not been elucidated. Therefore, we focused our work to assess the predictive value of PD-L1 expression in patients with bladder cancer. We also evaluated the link between PD-L1 expression and radiation response to provide new insights into the development of immune-based therapy.

## Results

### The expression of PD-L1 in human bladder cancer

**Figure 1:**
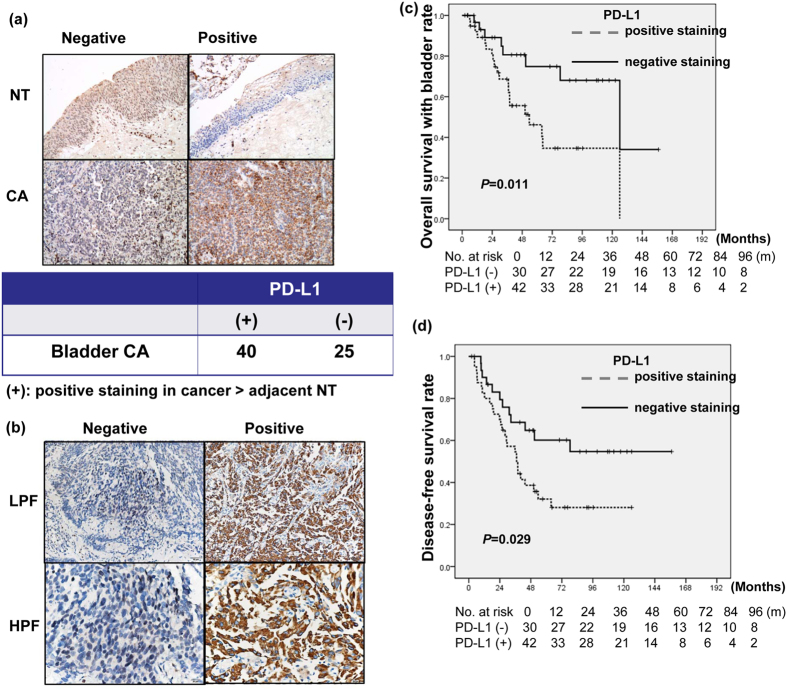
PD-L1 levels in bladder cancer correlated with clinical outcome. (**a**) Representative images of IHC staining with an anti-PD-L1 antibody of bladder cancer and adjacent non-malignant epithelium from TMA blocks (NT, adjacent non-malignant epithelium; CA, bladder cancer tissue). * (+) indicates positive staining in cancer > adjacent NT. (**b**) IHC staining with an anti-PD-L1 antibody of human bladder cancer specimens. Images of representative slides are shown at magnifications of ×100 (upper row) and ×200 (lower row). (**c**) Survival differences according to the staining of PD-L1 in overall survival with intact bladder; and disease-free survival (**d**) of bladder cancer patients treated with definite CCRT. The PD-L1-positive group exhibited reduced survival than the PD-L1-negative group.

Bladder tissue specimens retrospectively collected from 65 patients with MIBC (45 from TURBT at diagnosis and 20 from radical cystectomy) were constructed into TMA. IHC staining of TMA slides demonstrated that PD-L1 was overexpressed in the tumour tissues of 40 patients (61%) compared with adjacent non-malignant epithelial tissues (Fig. 1a). Figure 1b showed the representative slides of positive staining and negative staining with anti-PD-L1 antibody for human bladder cancer specimens at diagnosis. As listed in Table 1, of the 120 bladder cancer tissues, positive staining for PD-L1 was evident in 58% of T1-T4 bladder cancer tissues (27% (6/22) in T1, 47% (28/59) in T2 versus 72% (24/33) in T3-T4, P = 0.0003). There was a positive correlation between PD-L1 overexpression and cancers developing LN metastasis and loco-regional failure. Furthermore, Table 2 indicated that PD-L1 is a significant predictor of loco-regional recurrence on multivariate logistic regression for 92 patients with MIBC. Regarding the clinical data of 72 MIBC patients treated with definite CCRT, the 5yr OS, DFS, DSS and 5-yr survival with intact bladder is 64%, 44%, 75% and 58%, respectively. Table 3 and Fig. 1c,d demonstrated that the staining of PD-L1 was significantly linked with lower completed response rates, higher loco-regional failure rate and reduced DFS and lower survival with intact bladder for patients treated with definite CCRT. Although PD-L1 failed to reach statistical significance as a predictor of survival in the multivariate survival analysis, Table 4 demonstrated that the staining of PD-L1, not achieving complete response, and higher clinical stage were significantly associated with the risk of developing loco-regional recurrence analyzed by logistic regression. The findings suggested that PD-L1 contributes to treatment resistance in bladder cancer.

### Irradiation induced the expression of PD-L1 in bladder cancer *in vitro*

**Figure 2:**
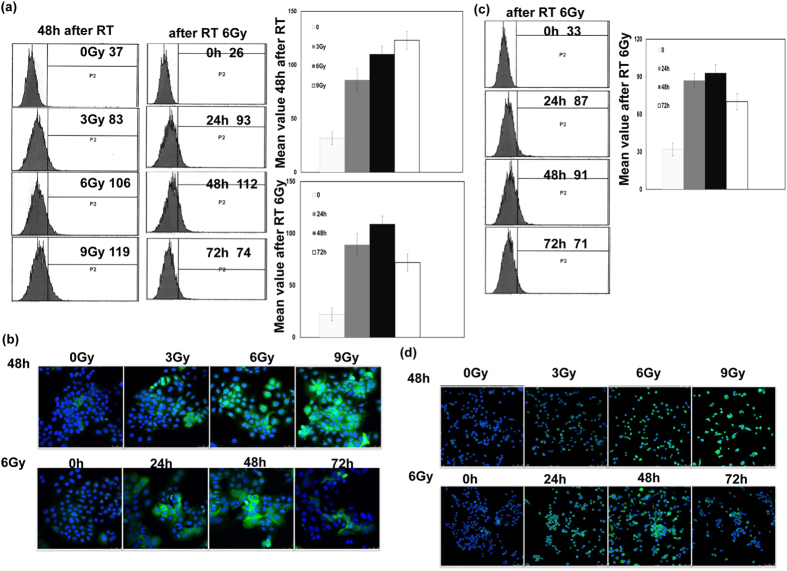
Effect of irradiation on PD-L1 expression in human bladder cancer. The levels of PD-L1 were evaluated by (**a**) FACS and (**b**) IF staining with the PD-L1 antibody for human bladder cancer cells, and (**c**,**d**) for murine bladder cancer cells at the indicated times after 6 Gy irradiation or at 48 h after RT with 0, 3, 6, and 9 Gy *in vitro*. Representative slides are shown.

We further examined the effect of irradiation on the expression of PD-L1 for bladder cancer *in vitro*. For human bladder cancer cell line HT1197, RT increased the expression of PD-L1 in tumour cells compared with nontreated cells, especially at 48h, and the increased levels of this protein were positively associated with the irradiation dose (Fig. 2a,b). Furthermore, as shown in Fig. 2c,d, PD-L1 expression in MB49 murine bladder cancer was significantly induced through irradiation when the cells were co-cultured with freshly isolated syngeneic CD11b+ cells^10^ from C57BL6 mice.

### PD-L1 correlates with radiation response of bladder cancer *in vivo*

**Figure 3:**
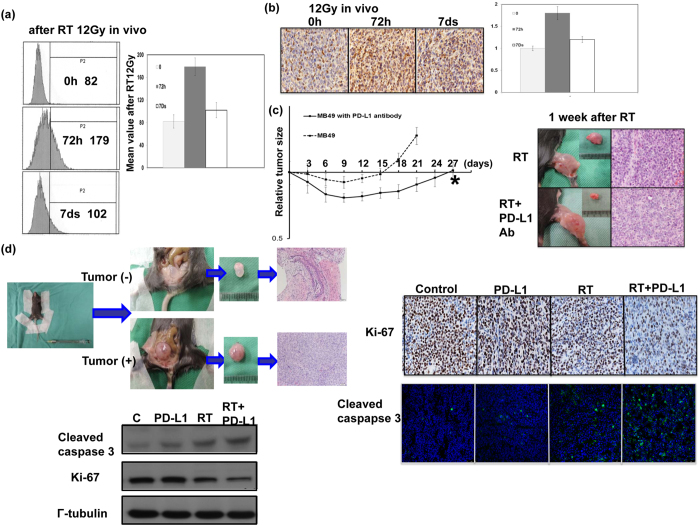
PD-L1 linked with the radiation response of murine bladder cancer *in vivo*. The effects of irradiation on PD-L1 expression *in vivo* were evaluated by (**a**) FACS and (**b**) IHC analyses using murine bladder tumour specimens at the indicated times following 12 Gy irradiation. Furthermore, effects of PD-L1 blockade on radiosensitivity *in vivo* were evaluated as the (**c**) growth delay of irradiated bladder tumours. The Y- axis shows the ratio of tumour volumes at each time point divided by that at irradiation, respectively. Each point is shown as the means of 3 separate experiments; bars, SD. ^*^P < 0.05; (**d**) using an orthotopic murine tumour model, IHC for tumour cell proliferation rate at 7 days after irradiation; immunofluorescence for apoptosis at 72 h after irradiation; and western blot analysis for ki-67 and cleaved caspase 3 in tumours at indicated time after irradiation. Representative slides are shown.

As shown in Fig. 3a,b, the expression of PD-L1 in the irradiated ectopic tumours confirmed the *in vitro* findings. This radiotherapy-mediated increase in tumour cell PD-L1 expression peaked at 72 hours after radiotherapy and significantly declined at 7 days after radiotherapy. To determine whether PD-L1 is associated with bladder cancer radiosensitivity *in vivo*, MB49 tumour cells were implanted, and 14 days later, the tumours were treated with RT (12 Gy) with or without anti–PD-L1. The results shown in Fig. 3c demonstrated that anti–PD-L1 significantly sensitized the MB49 bladder tumour to irradiation. The growth delay of RT alone and RT plus anti–PD-L1 was 15.23 +/− 0.79 and 26.96 +/− 0.92 days, respectively. Furthermore, Fig. 3d demonstrated that that PD-L1 blockade decreased cell proliferation and augmented cell death after irradiation using an orthotopic tumour model with Ki-67 and cleaved caspase 3 staining.

### PD-L1 increased radio-resistance through the inhibition of CD8+ T cells

**Figure 4:**
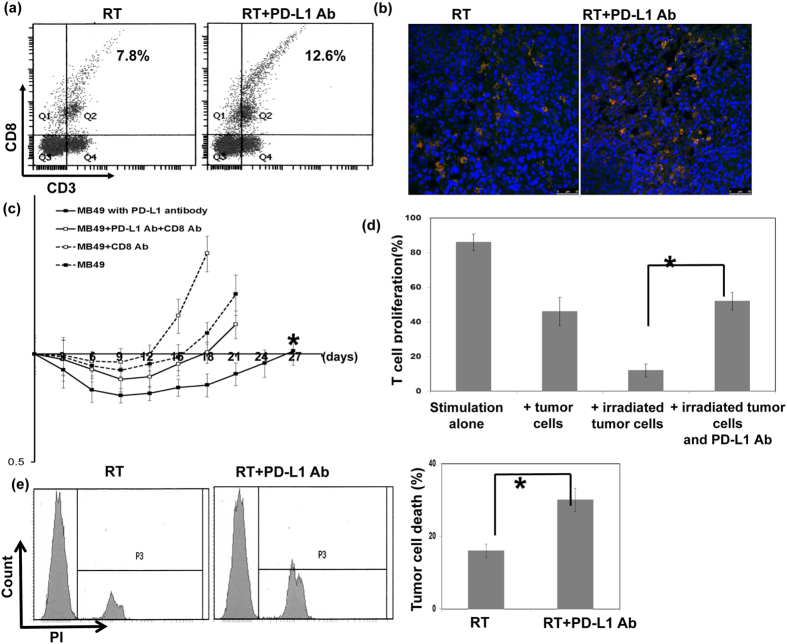
Correlation between irradiation, PD-L1, and cytotoxic T cell accumulation. The effect of PD-L1 blockade on tumour-infiltrated cytotoxic T cells was evaluated by (**a**) FACS using CD3-CD8 and (**b**) immunofluorescence analysis (DAPI, blue; CD3, Red; and CD8, Green) using irradiated tumour specimens. (**c**) The effect of CD8+ T cells on PD-L1 Ab-induced radiosensitization was further evaluated in irradiated mice treated with or without CD-8 neutralizing antibodies using tumour growth delay after irradiation. (**d**) The effect of PD-L1 blockade on the suppressing ability of tumor cells for T cells proliferation was evaluated by FACS. Quantitative data are shown. (**e**) The effect of PD-L1 blockade on the CD8+ T cells cytotoxicity against irradiated cancer cells was evaluated by FACS. Representative images and quantitative data are shown.

We analysed the recruitment of CD8+ T cells in tumours using irradiated mice treated with or without anti-PD-L1. The data in Fig. 4a,b showed that the PD-L1 blockade increased the infiltration of CD8+ T cells in irradiated tumours. The depletion of CD8+ T cells significantly abolished the effectiveness of the combination treatment, resulting in rapid tumour outgrowth (Fig. 4c).To further test the functional consequences of up-regulation of PD-L1 in tumor cells-mediated T cell suppression, the function of T cells against tumor cells was evaluated with or without blocking PD-L1. Irradiation increased the ability of tumor cells to suppress nonspecific stimuli (anti-CD3/CD28 antibody)-mediated T cell proliferation, and anti-PD-L1 attenuated the ability of T cell suppression induced by irradiated tumor cells (Fig. 4d). Inhibition of PD-L1 combined with irradiation resulted in increased tumor cytolysis compared with irradiation alone when tumor cells co-cultured with sorting CD8+ cells from tumor-bearing mice (Fig. 4e).

## Discussion

Malignant tumors possess mechanisms for evading host immune responses. A novel mechanism that tumor may evade host immune response through the expression of PD-L1. PD-L1 expression has been observed in a wide variety of solid malignancies, and might be correlated with the higher malignant grade of tumours, increased tumour growth, and the patients’ prognoses^10^,^19^,^20^,^21^. Identification of potential factors that can stratify a tumor’s response to specific therapies will aid in the selection of bladder cancer therapy, including whether bladder-sparing approach like CCRT is warranted. To investigate the clinical significance of PD-L1 in bladder cancer, we studied the role of PD-L1 in treatment response and its predictive value for prognosis in the present study. We examined the link between the level of PD-L1 and clinical characteristics in bladder cancer. The overexpressed PD-L1 was significantly associated with clinical tumor stage and positive lymph node metastasis. Furthermore, for MIBC patients treated with definite CCRT, enhanced expression of PD-L1 was significantly associated with a lower complete response rate after treatment, a higher disease failure rate and a shorter survival with intact bladder. The data demonstrated a role of PD-L1 in predicting prognosis and the treatment efficiency of CCRT for bladder sparing. By multivariate analysis, the staining of PD-L1 possessed the ability to predict the risk of loco-regional recurrence. In addition to clinical T stage, regional LN status has been reported as an independent predictor for bladder cancer. However, our clinical data could not demonstrate the clinical significance of LN involvement. It might be due to the limited number of patients with LN involvement and heterogeneity in staging and treatment in the present study.

Emerging evidence suggests that the generation of antitumor immune responses might play an important role in the effectiveness of radiotherapy^22^,^23^. PD-L1, a major molecular regulator of tumour immune escape, inhibits T cell-mediated immune attack by binding to the PD-1 receptor on tumour-specific T cells^9^,^10^. PD-L1 has been reported to associate with the resistance to anticancer therapies^11^,^12^. We observed that irradiation increased PD-L1 expression of bladder cancer cells *in vitro*. The upregulation of PD-L1 was also observed in irradiated tumours in mice, suggesting that the alteration of PD-L1 levels in tumour microenvironments might play a role in the radiation response of bladder cancer. To address this issue, we combined irradiation with anti-PD-L1 antibody therapy to treat murine MB49 bladder cancer in immunocompetent animal models. The data revealed that the anti-PD-L1 antibody sensitized bladder cancer to radiation, as demonstrated by a longer tumour growth delay for ectopic tumors. Furthermore, by mouse orthotopic models, blocking PD-L1 augmented the RT-induced tumour cell death associated with decreased tumor cell proliferation compared with tumours after radiation therapy alone.

Radiation increased the T cell recognition of irradiated tumour cells, making the cells vulnerable to cytotoxic T lymphocyte-mediated clearance^24^,^25^. The upregulation of the PD-1/PD-L1 axis suppressed the cytotoxic action of T cells, likely reflecting the observed incomplete tumour cell killing after irradiation^12^. The blockade of PD-L1 is an effective strategy for partially restoring T cell function^26^,^27^. Therefore, we investigated whether the radiosensitization induced through anti-PD-L1 therapy is associated with restoring the functionality of cytotoxic T cells. The analysis of CD8+ T cells in a mouse bladder model revealed that anti-PD-L1 increased CD8+ T cell infiltration in irradiated tumours. Furthermore, depletion experiments indicated that CD8+ T cells were required for the efficacy of the combined radiation and PD-L1 blockade therapy. PD-L1 is thought to limit T-cell function within tissue sites, including cell proliferation and the cytotoxicity induced by T cells^28^,^29^. Therefore, we investigated the role of PD-L1 in the functionality of cytotoxic T cells *in vitro*. The data revealed that tumor cells’ PD-L1 expression was triggered by radiation. Blockade of PD-L1 effectively inhibit CD8+ T cells cytotoxicity against irradiated bladder cancer cells. Furthermore, we demonstrated that the increase in PD-L1 expression on irradiated bladder cancer cells was associated with a decreased T cell proliferation upon co-culture experiments, and anti-PD-L1 had reduced the suppressive ability for T cell proliferation. Thus, these results showed that PD-L1 expression negatively correlated with the numbers and function of cytotoxic T cells, which linked to tumor irradiation response demonstrated by the immunocompetent animal model. Therefore, we suggested that PD-L1 affects the radiation response of bladder cancers mediated by regulating the tumor microenvironment in the immunocompetent host.

For the interpretation of our results, different limitations have to be taken into account. First, we examined PD-L1 expression mainly in biopsy tissue specimen from TURBT to evaluate its prognostic value. Second, the patient population enrolled in the retrospective study are heterogeneous with relatively small group sizes. Thus, further investigations that included more patients in a prospective trial are needed.

## Conclusions

The ultimate cancer treatment would leverage different cytotoxic pathways for the complete eradication of tumours. The data indicated that PD-L1is a predictor of treatment response and loco-regional recurrence for bladder cancer. Therefore, measured PD-L1 expression levels in bladder cancer excision tissues provides further data stratifying patients to higher risk disease and allow selection for personalized treatment.

## Materials and Methods

### Patient characteristics and tissue specimens

This study was approved by the Institutional Review Board of Chang Gung Memorial hospital, and performed in accordance with the approved guidelines. Informed written consent for the acquisition and storage of medical information and tissue specimens was obtained from all of the patients. There were 72 patients with histopathological proven muscle invasive urothelial cell carcinoma who completed definite chemoradiotherapy (CCRT) treatment enrolled in the study. On completion of definite CCRT, patients underwent abdomen CT, cystoscopy and urine cytology to assess the treatment response and disease status every 3 to 4 months for the first 2 years, and then every 6 months during follow-up. The median follow-up time was 3.8 years. Clinical CR was defined as no tumor visible on cystoscopy, negative tumor site biopsy, and negative urine cytology after the completion of CCRT. The identification of clinical lymph node (LN) involvement with CT was based on measurements of node size, with greater than 1 cm being the criterion. Specimens were retrospectively collected from the 72 patients for immunochemical analysis. The end points were overall survival (OS), disease-free survival (DFS) and the response to definite CCRT. The loco-regional recurrence was defined by tumor progression in bladder or pelvic nodes assessed with CT and cystoscopy. Survival probabilities were analyzed using the Kaplan-Meier method.

### Immunohistochemical (IHC) staining

Formalin-fixed, paraffin-embedded bladder tissues collected from TURBT at diagnosis and/or radical cystectomy for bladder cancer were subjected to immunochemical analyses. These 120 urothelial cell carcinoma patients in Table 1 included 72 patients with clinical stage T2–T4 treated with definite CCRT, 20 with pathologic stage T2 bladder cancer treated with radical cystectomy and pelvic lymph node dissection, and 28 patients with clinical stage T1 NMIBC (tumor confined in lamina propria) were subjected to IHC staining. Of the 20 patients treated with radical cystectomy, the LN status was assessed by pathologic findings with a median of 10 (range 4–26) nodes removed. Among these tissue specimens, 65 bladder tissues were converted into tissue microarray (TMA) blocks contained bladder cancer and the adjacent non-malignant epithelium using an AutoTiss 1000 arrayer (Ever BioTechnology, Canada). In cases in which blocks were available, a pathologist re-evaluated the slides stained with haematoxylin and eosin to assess the quality of the TMA slides. Formalin-fixed, paraffin-embedded tissues were cut into 4-μm sections, mounted on slides, deparaffinized with xylene and dehydrated using a graded ethanol series. The slides were incubated overnight at 4 °C using antibodies against PD-L1. The IHC data for the specimens were assessed using the semi-quantitative immunoreactive score (IRS). The IRS was calculated by multiplying the staining intensity (graded as follows: 0 = no, 1 = weak, 2 = moderate and 3 = strong staining) and the percentage of positively stained cells (0 = less than 10% of stained cells, 1 = 11–50% of stained cells, 2 = 51–80% of stained cells and 3 = more than 81% of stained cells). An ROC curve was calculated and best cut-off points were determined in bladder cancer cells compared with adjacent non-malignant tissue. An IRS scoring grade of >=2 was considered positive IHC scoring

### Cell culture and reagents

HT1197, a human bladder cancer cell line, was obtained from the American Type Culture Collection (ATCC). We maintained the bladder cancer cells in DMEM supplemented with 10% foetal bovine serum. Mouse bladder carcinoma MB49 cells were kindly gifted from Dr Yi-Wen Liu^30^. The MB49 cells were maintained in RPMI 1640 medium supplemented with 10% FBS. No further authentication was conducted for the two cancer cell lines.The PD-L1-neutralizing antibody (human and mouse) were obtained from Biolegend (San Diego, CA). Antibodies to Ki-67 protein, a cellular marker of proliferation, and to cleaved caspase 3, an indicator of cell apoptosis, were obtained from Santa Cruz Biotechnology, Inc. (Santa Cruz, CA).

### Ectopic and orthotopic tumour models

This study was performed in strict accordance with the recommendations in the Guide for the Care and Use of Laboratory Animals as promulgated by the Institutes of Laboratory Animal Resources, National Research Council, USA. The protocol was approved by the Committee on the Ethics of Animal Experiments of Chang Gung Memorial Hospital. Eight-week-old female C57 mice were used as the tumour implantation model. To investigate the link between PD-L1 expression and the radiation response in immunocompetent host^31^, we examined the radiation response of MB49, the corresponding syngeneic TCC cell line in C57/BL6 mice, *in vivo*. In the ectopic tumour implantation model, 1 × 10^6^ tumour cells were subcutaneously implanted by injection into the dorsal gluteal region (five animals/group). In the orthotopic tumour implantation model, we performed intravesical instillation of cancer cells as previously described^32^. To determine the radiosensitivity *in vivo*, local irradiation to 12 Gy was performed when the ectopic tumours reached 0.5 cm^3^ or at 2 weeks after orthotopic tumour implantation. Radiosensitivity was indicated as growth delay (*i*.*e*., the time required for the tumour to recover the previous volume after irradiation). Duplicate experiments were performed for growth delay analyses. To evaluate the effect of PD-L1 blockade on the radiation response and immune cells *in vivo*, the intraperitoneal (i.p.) injection of a PD-L1 neutralization antibody (250 μg per mouse, two times per week) was initiated at 1 day prior to irradiation and continued for 2 weeks.

### Flow cytometry

To obtain single-cell suspensions, the tumour tissues were digested using 1 mg/ml collagenase IV (Sigma-Aldrich) and 0.2 mg/ml DNase I (Sigma-Aldrich) for 45 minutes at 37 °C. The cells were blocked and subsequently stained with antibodies against CD8, CD3, and PD-L1. For cytotoxicity assays, tumor cells were stained with PI after the removal of CD8+ cells. The murine cytotoxic T cells from fresh tumour specimens were characterized and sorted as CD3+CD8+.

### T-cell Suppression Assay

The negative regulation of tumour-infiltrating T cells through PD-L1/PD-1 might be an important host-mediated mechanism of acquired radioresistance in tumours^33^,^34^.The suppressive function of tumor cells was measured as the inhibition of autologous T cell proliferation using a suppression assay as previously described^35^. The isolated T cells were CFSE-labelled (3 μM, Sigma) and seeded in 96-well plates (at 2 × 10^5 ^cells/well), and tumors were previously isolated at a 2:1 ratio. T cell proliferation was induced using anti-CD3/CD28 stimulation beads (Invitrogen, Carlsbad, CA). After three days, the suppression assay was analysed for T cell proliferation using flow cytometry.

### Statistical analysis

The significance of the differences between the samples was determined using Student’s t-tests. The data are presented as the means ± standard error of the mean (SEM). All experiments, comprising three replicates, were performed at least twice independently. A probability level of p < 0.05 was considered statistically significant, unless otherwise stated.

## Additional Information

**How to cite this article**: Wu, C.-T. *et al.* The role of PD-L1 in the radiation response and clinical outcome for bladder cancer. *Sci. Rep.* **6**, 19740; doi: 10.1038/srep19740 (2016).

## Figures and Tables

**Table 1:** Clinico-pathological characteristics of 120 bladder cancer patients.

	**No. of patients**		***p*****value**
	**PD-L1 (−)**	**PD-L1 (+)**	
Age			0.415
Median	71	73.1	
Range	45.5–91.5	46.5–90	
Gender			0.39
Male	49 (79%)	42 (72%)	
Female	13 (21%)	16 (28%)	
T-stage			0.0003^*^
T1	22 (35%)	6 (10.3%)	
T2	31 (50%)	28 (48.3%)	
T3-T4	9 (15%)	24 (41.4%)	
Pathologic grade			0.83
Low-intermediate	33 (53%)	32 (55%)	
High	29 (47%)	26 (45%)	
LN involvement			0.013^*^
Negative	51 (82%)	36 (62%)	
Positive	11 (18%)	22 (38%)	
Distant metastasis			0.14
Negative	56 (90%)	47 (81%)	
Positive	6 (10%)	11 (19%)	
Loco-regional failure			
Negative	55 (89%)	25 (43%)	0.000^*^
Positive	7 (11%)	33 (57%)	

**Table 2:** Analysis to determine factors associated with the risk of loco-regional recurrence.

**Variables**	**Odd ratios**	**95% confidence interval**	**p**
PD-L1 staining	0.093	0.029–0.301	0.000*
Clinical stage (T2 vsT3-T4)	3.109	0.929–10.403	0.066
Treatment (surgery vs CCRT)	6.409	1.286–28.453	0.023*
LN involvement	0.613	0.205–1.828	0.380

**Table 3:** Clinico-pathological characteristics of patients with MIBC treated with definite CCRT.

	**No. of patients**		***p*****value**
	**PD-L1 (−)**	**PD-L1 (+)**	
Age			0.64
Median	71.8	73.3	
Range	48.5–90.2	46.5–88.2	
Gender			0.61
Male	22 (73%)	32 (76%)	
Female	8 (27%)	10 (24%)	
Pathologic grade			0.16
Low-intermediate	15 (50%)	28 (67%)	
High	15 (50%)	14 (33%)	
Clinical stage			0.005^*^
T2	21 (70%)	18 (43%)	
T3-T4	9 (30%)	24 (57%)	
Clinical LN involvement			0.066
Negative	24 (80%)	25 (60%)	
Positive	6 (20%)	17 (40%)	
RT dose (cGy)			0.29
mean	5819	5820	
median	6000	5940	
Response to			
definite CCRT			
CR (+)	27 (90%)	24 (57%)	0.002^*^
CR (−)	3 (10%)	18 (43%)	
Distant metastasis			0.55
Negative	24 (80%)	32 (76%)	
Positive	6 (20%)	10 (24%)	
Loco-regional failure			
Negative	23 (77%)	15 (36%)	0.000^*^
Positive	7 (23%)	27 (64%)	
Survival rate			
5yr OS	75%	55%	0.113
5yr DFS	60%	32%	0.029^*^
5yr DSS	88%	65%	0.032^*^
5yr survival with bladder	74%	46%	0.011^*^

Abbreviations: CR = complete response; OS = overall survival; DFS = disease-free survival;

**Table 4:** Factors associated with the risk of loco-regional recurrence for patients with MIBC treated with definite CCRT.

**Variables**	**Odd ratios**	**95% confidence interval**	**p**
PD-L1 staining	0.164	0.047–0.377	0.005*
Clinical stage (T2 vs T3–T4)	3.817	1.026–14.209	0.046*
Treatment response (CR or not)	5.366	1.317–21.858	0.019*
Clinical LN involvement	0.409	0.125–1.337	0.139
